# Recipient Survival among Living Donor vs. Deceased Donor Liver Transplants for Acute Liver Failure in the United States

**DOI:** 10.3390/jcm13061729

**Published:** 2024-03-17

**Authors:** Eric Moughames, Merve Gurakar, Amir Khan, Marwan Alsaqa, N. Begum Ozturk, Alan Bonder, Ahmet Gurakar, Behnam Saberi

**Affiliations:** 1 Division of Gastroenterology and Hepatology, Johns Hopkins University School of Medicine, Baltimore, MD 21287, USA; 2 Johns Hopkins University School of Public Health, Baltimore, MD 21205, USA; 3 Beth Israel Deaconess Medical Center, Harvard Medical School, Division of Gastroenterology and Hepatology, Boston, MA 02215, USA; 4 Department of Internal Medicine, Beaumont Hospital, Royal Oak, MI 48073, USA

**Keywords:** acute liver failure, living donor liver transplantation, deceased donor liver transplantation

## Abstract

**Objectives:** Acute liver failure (ALF) is associated with high morbidity and mortality. Timely liver transplantation (LT) is the only universally accepted therapy for ALF that is non-responsive to medical therapy. Data regarding the use of living donor LT (LDLT) for this indication in the US is scarce. **Materials and Methods**: United Network of Organ Sharing/Organ Procurement and Transplantation Network (UNOS/OPTN) data from January 2002 to December 2020 were reviewed. Adult and pediatric recipients listed as status 1 were included. Demographics, clinical and laboratory data, and post-LT survival rates were compared for LDLT vs. DDLT recipients. **Results**: There were 180 LDLT (3.6%) and 4779 DDLT (96.4%) recipients with a diagnosis of ALF. The majority of recipients in the LDLT group were pediatric (n = 164, 91%) compared to the DDLT group (n = 1455, 30%), *p* < 0.001. In the pediatric-only group, post-LT survival was comparable between LDLT and DDLT recipients (*p* = 0.15). Five-year post-LT survival was higher for pediatric recipients compared to adults in the LDLT group (84.2% vs. 62.5%, respectively, *p* < 0.001) and the DDLT group (82.8% vs. 78.7%, respectively, *p* < 0.001). Adults had a higher hazard of death compared to pediatric recipients in the LDLT group (HR = 3.560, 95% CI 1.612–7.844, *p* = 0.002) and the DDLT group (HR = 1.472, 95% CI 1.290–1.679, *p* < 0.001). In multivariate analysis results, the type of LT and age group were not associated with higher post-LT mortality. **Conclusions**: In the US, LDLT constitutes 3.6% of LTs for ALF. In the pediatric-only group, post-LT survival was comparable between LDLT and DDLT recipients. Overall, there were superior post-LT outcomes for pediatric recipients compared to adults for LDLT and DDLT.

## 1. Introduction

Acute liver failure (ALF) is a life-threatening condition defined by the development of hepatic encephalopathy and coagulopathy that is characterized by an international normalized ratio (INR) ≥ 1.5 and often the development of multi-organ failure leading to death in a patient with no pre-existing liver disease [1,2]. Despite improvements in survival rates due to more sophisticated supportive care methods in the intensive care unit, emergent liver transplantation (LT) remains the only treatment for patients who do not improve [3,4,5].

Deceased donor LT (DDLT) and living donor LT (LDLT) have been utilized to treat ALF, and the rate and type of LT depend on the country in which they are performed [6,7,8,9]. DDLT is the most common type of LT in the United States (US), Canada, Europe, and Australia due to these countries’ more efficient deceased donor allocation systems compared to those of the rest of the world [10,11,12]. In countries with limited resources and infrastructure and where deceased donation is scarce, LDLT is more prevalent and is often the first-line treatment option for ALF [13,14,15,16,17]. Despite an efficient deceased donor system in the US, an increasingly busy waitlist and long waiting times have led many centers to expand their donor selection to include LDLTs; however, data regarding the utilization and efficacy of LDLTs for ALF have been conflicting [7,18,19,20].

In this study, we used data from the United Network for Organ Sharing (UNOS) and the Organ Procurement and Transplantation Network (OPTN) and aimed to investigate the characteristics and outcomes of patients with ALF who received LDLT compared to those who received DDLT in the US.

## 2. Materials and Methods

### 2.1. Data Source

The information reported was sourced from the UNOS, which acts as a contractor for the OPTN in the US. The UNOS is a non-profit organization that works in conjunction with the federal government to manage the transplant system in the US [1]. Any conclusions or interpretations made in this article are solely the responsibility of the authors and should not be considered official policies or views of the OPTN or the US government. No Institutional Review Board approval was required for this data source or this study.

### 2.2. Study Design and Patient Population

Data was collected for all patients, adult and pediatric, diagnosed with ALF who underwent LDLT or DDLT from January 2002 to December 2020, using the diagnostic code for END_STAT as 6010 and 6011, listed as status 1 or 1A in the UNOS database. Status 1/1A includes patients who have ALF and are unlikely to survive for more than 7 days without LT. Pediatric patients with status 1B were excluded from this study, as these candidates had underlying chronic liver disease. Data regarding gender, age, and race/ethnicity, as well as laboratory data (regarding INR, bilirubin, and creatinine) and clinical data, such as those regarding ascites and hepatic encephalopathy, were collected.

### 2.3. Study Outcome

The primary outcome of this study was survival (years) after LT. Post-LT survival was analyzed for LDLT and DDLT groups. Separate analyses were performed for adult and pediatric LT recipients. 

### 2.4. Statistical Methods

Stata 16.1 was used for statistical analysis. Categorical variables are reported as numbers and percentages, and continuous variables are reported as means and standard deviations.

The chi-square test was applied to compare categorical variables, while the Kruskal–Wallis test was applied to compare continuous variables, and *p*-values were calculated.

Kaplan–Meier curves were plotted to estimate survival outcomes for both groups. The log-rank test was applied to test whether the two groups had significant differences in survival outcomes. Forward stepwise multivariate Cox regression analyses were completed to identify significant predictors of survival, adjusted for recipient and donor characteristics, where the *p*-value for entry into the model was set at 0.05, and a *p*-value of 0.1 was set for removal from the model. Clinically relevant and statistically significant variables were entered into this stepwise model. Variables included in the model were age, gender, race, blood type, ascites, encephalopathy, serum creatinine, INR, bilirubin, sodium, dialysis, life support, adult vs. pediatric, DDLT vs. LDLT, donor age, donor gender, donor BMI, and donor blood type. A hazard ratio (HR) represented a 95% confidence interval (CI). No comparisons were made between adult LDLT vs. DDLT patients, given the small number of patients who underwent LDLT. Statistical significance was defined as *p* < 0.05 for all parameters.

## 3. Results

### 3.1. Characteristics of Pediatric LDLT vs. DDLT Recipients with ALF

There were 180 patients who underwent LDLT for ALF. Among these, 164 (91.1%) were pediatric LT recipients. In the DDLT group there were 4779 patients; 1455 (30.5%) were pediatric LT recipients. Table 1 compares LDLT vs. DDLT for ALF for pediatric-only recipients and their donors. There were 1619 pediatric LT recipients for status 1A ALF from 2002 to 2020 in the US (Table 1). Of these, 1,455 (89.9%) underwent DDLT and 164 (10.1%) underwent LDLT. Patients who received LDLT were younger (median age 3 vs. 4 years, *p* < 0.001) and more likely to be male compared to those who received DDLT (*p* = 0.04). In addition, the proportion of African American/Black recipients was significantly lower in the LDLT group compared to the DDLT group (11.6% vs. 17.5%, respectively, *p* = 0.02). Patients who received LDLT were less likely to have ascites or hepatic encephalopathy (*p* < 0.001 and *p* = 0.04, respectively), and less likely to receive dialysis (*p* = 0.02). Patients who received LDLT had significantly lower wait times compared to patients who received DDLT (median 3.5 vs. 5 days, respectively, *p* = 0.01). Donors in the LDLT group were older and more likely to be females compared to donors in the DDLT group (*p* < 0.001). The cold ischemia time was shorter in the LDLT group compared to DDLT group (*p* < 0.001). Table 2 summarizes the descriptive analysis of adult LDLT and DDLT cases. There were only 16 patients with diagnostic codes for ALF status 1 who received a LDLT.

### 3.2. Survival Analysis

#### 3.2.1. Survival Comparison of All ALF Patients Who Received LDLT vs. DDLT

**Figure 1:**
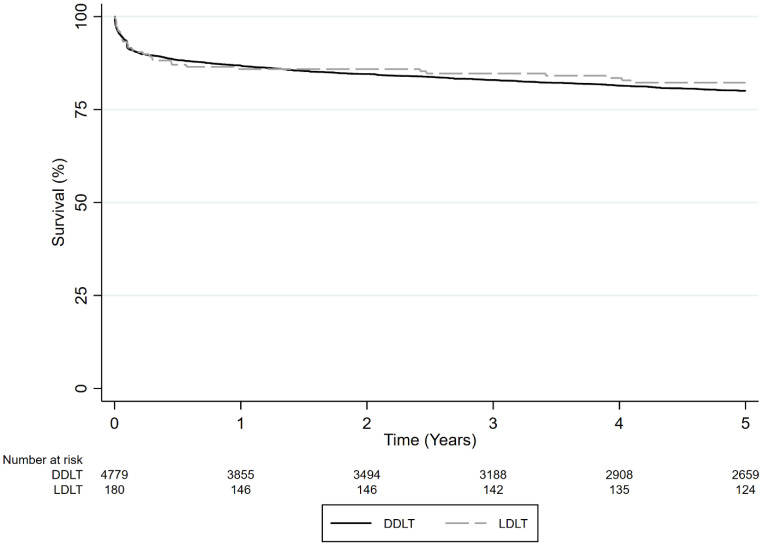
Survival comparison of LDLT vs. DDLT in adult and pediatric LT recipients with ALF (*p* = 0.02).

Overall, 1-, 3-, and 5-year survival rates were higher for LDLT compared to DDLT in adult and pediatric patients combined; LDLT survival rates were 85.9%, 84.7%, and 82.2%, respectively, vs. DDLT survival rates of 86.8%, 82.9%, and 80.0%, respectively (*p* = 0.02) (Figure 1). In univariate analysis results, DDLT was associated with a higher hazard of death compared to LDLT (HR = 1.479, 95% CI 1.057–2.071, *p* < 0.02). In univariate analysis results, adults had a higher hazard of death compared to pediatric patients (HR = 1.518, 95% CI 1.337–1.723, *p* < 0.001). However, in multivariate analysis results, the type of LT and age group were not associated with higher mortality.

#### 3.2.2. Survival of Pediatric ALF Patients Who Received LDLT vs. DDLT

**Figure 2:**
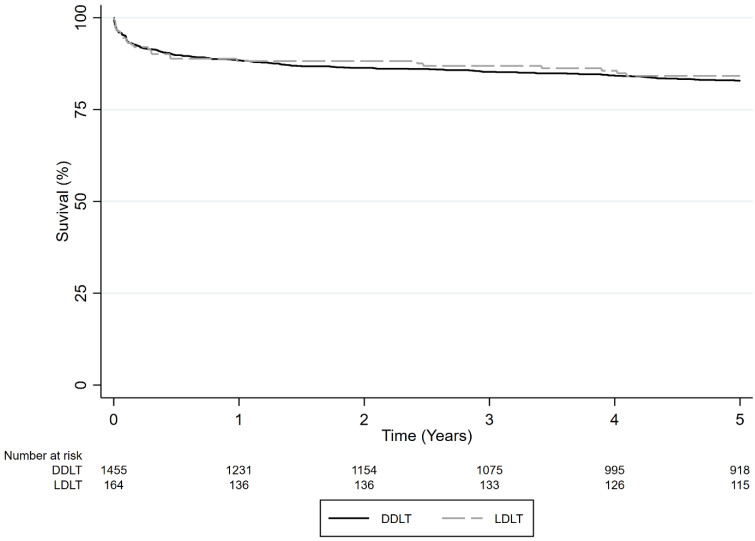
Survival comparison of LDLT vs. DDLT in pediatric recipients with ALF (*p* = 0.15).

In the pediatric-only group, 1-, 3-, and 5-year survival rates were comparable between patients who underwent LDLT ( 88.2%, 86.9%, and 84.2%, respectively) and DDLT (88.4%, 85.3%, and 82.8%, respectively) (*p* = 0.15) (Figure 2). In univariate analysis results, DDLT was not associated with a higher hazard of death compared to LDLT in pediatric patients (HR = 1.338, 95% CI 0.902–1.983, *p* = 0.15). In multivariate analysis results, DDLT was not associated with higher mortality.

#### 3.2.3. Survival Comparison of Adult vs. Pediatric Patients Who Received LDLT for ALF

**Figure 3:**
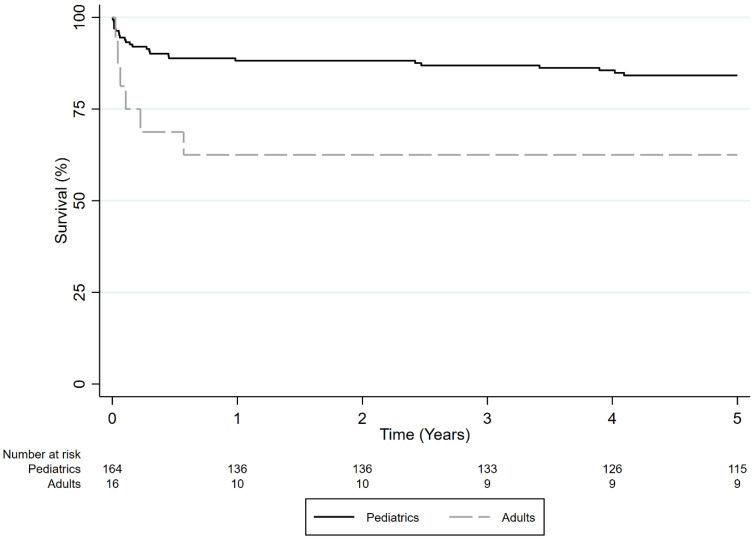
Survival of adult vs. pediatric LDLT recipients with ALF (*p* < 0.001).

Overall, 5-year survival rates were significantly higher in pediatric patients compared to adults with ALF who underwent LDLT (84.2% and 62.5%, respectively) (*p* < 0.001) (Figure 3). In univariate analysis results, adults had a higher hazard of death compared to pediatric patients in the LDLT group (HR = 3.560, 95% CI 1.612–7.844, *p* = 0.002). However, only 16 adult patients with ALF received LDLT. In multivariate analysis results, the adult group that underwent LDLT was not associated with higher mortality compared to pediatric patients.

#### 3.2.4. Survival of Adult vs. Pediatric Patients Who Received DDLT for ALF

**Figure 4:**
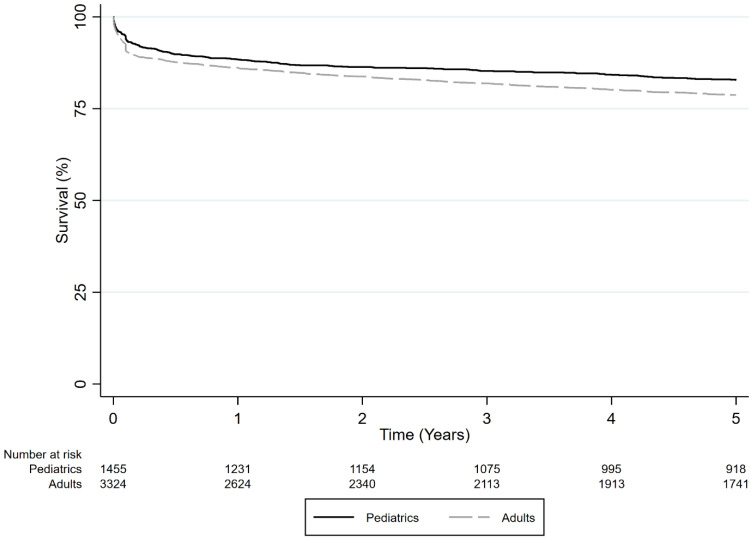
Survival comparison of adult vs. pediatric DDLT recipients with ALF (*p* < 0.001).

Overall, 1-, 3-, and 5-year survival rates were higher for pediatric patients (88.4%, 85.3%, and 82.8%, respectively) compared to those for adults (86.1%, 81.9%, and 78.7%, respectively) with ALF who underwent DDLT (*p* < 0.001) (Figure 4). In univariate analysis, adults had a higher hazard of death compared to pediatric patients in the DDLT group (HR = 1.472, 95% CI 1.290–1.679, *p* < 0.001). However, in multivariate analysis the adult DDLT age group was not associated with higher mortality.

## 4. Discussion

There was overall improved survival among patients who underwent LDLT as compared to DDLT for ALF in adult and pediatric recipients combined; however, in the pediatric population there was no difference in survival between patients who underwent LDLT and DDLT for ALF. Our study also suggests that, overall, pediatric patients had a better survival rate irrespective of the type of LT (LDLT or DDLT) compared to adults; however, it is important to note that the total number of LDLTs in adult patients was small.

DDLT continues to be the leading type of LT in the US, especially for patients with ALF that are listed as status 1 or 1A [21]. However, due to more recent international and local studies published regarding donor safety and recipient survival, there has been an increase in LDLT in the US over the past decades, and multiple publications have reported patient outcomes to be similar for DDLT and LDLT [22,23,24,25,26]. In addition, the US population is gradually aging, with 15% of the population expected to be above 70 years old by 2030, which both increases the number of patients on the waitlist and decreases the donor pool, as the majority of LT centers in the US currently decline livers for DDLT from donors older than the age of 60 [27,28,29].

Our study demonstrated improved 1-, 3- and 5-year survival rates for LDLT compared to DDLT in patients with ALF. Our results show significantly higher survival rates compared to those of a study by Urrunaga et al., which compared post-LDLT and post-DDLT patient survival in the US and showed no difference in 1- and 5-year survival rates between the two patient groups [30]. In Urrunaga et al.’s 2014 study, 1-year survival rates for LDLT and DDLT were 71% and 79%, respectively, and the 5-year survival rate for both LDLT and DDLT was 71% [30]. These numbers are lower than our current findings of 87% survival at year 1 for LDLT and 85.5% for DDLT, and 81.1% 5-year survival for both LDLT and DDLT. This improvement in survival rate over the last decade parallels the increase in the number of LDLTs performed in the US [22,23,24]. This can also be explained by improvements in surgical techniques, organ preservation, and graft survival over the past decade [31].

We found that the presence of ascites, hepatic encephalopathy, and the need for dialysis were all significantly less common in patients who underwent LDLT compared to those of patients who underwent DDLT. Similarly, another study reported that patients with primary sclerosing cholangitis who underwent LDLT less commonly had ascites and hepatic encephalopathy compared to those whose underwent DDLT, although this study was not conducted in patients with ALF [32].

We also found that the majority of LDLTs performed involved pediatric patients (93.5%). This was due to high pediatric waitlist mortality and a small pool of deceased donors, likely due to size discrepancies, compared to adult LTs [33,34,35,36]. When comparing LDLT to DDLT in the pediatric population alone, we found that there was no difference in survival at one, three, and five years. This is consistent with previous studies that demonstrated similar outcomes in pediatric populations [37]. A study by Firl et al. showed no statistical differences in overall patient survival between graft types [37]. This non-inferiority could be due to multiple factors, including a usually shorter waiting time for LDLT recipients compared to DDLT recipients. It has also been argued that having a robust social support system can increase a patient’s chances of finding a living donor, providing lifesaving LT and a shorter wait time. In addition, this usually coincides with improved compliance and access to healthcare services, leading to good outcomes [38].

One of the important observations from our study was that African American/Black recipients with ALF were less frequently represented in the LDLT group, and that needs to be further explored. A study conducted regarding the utilization of LDLT reported that African American/Black patients received lower per-patient donation inquires compared to White patients, however this study excluded patients with ALF [39].

Our study also demonstrated higher survival rates in pediatric patients receiving DDLT compared to adult patients at each time point measured. These findings have important clinical implications, as they suggest that age is a crucial factor in predicting outcomes for patients receiving DDLT. The higher survival rates observed in pediatric patients may be due to several factors, including their relatively better health status, with a lower incidence of comorbidities compared to adult patients, and a higher regenerative potential of the liver [40,41].

Finally, it has been reported that living donor evaluation and preparation can take approximately 36–40 h, which can limit the number of eligible living liver donors [4]. Such time-line requirements and lack of institutional protocols and experience required to serve the rapid evaluation process of donors may further limit the usage of LDLT as an option for adult patients with ALF.

This study had several limitations, and it should be interpreted within its context. First, only a small number of patients underwent LDLT compared to DDLT, especially adult recipients. This restricted performance of a comparison between the two groups. Second, although we adjusted for laboratory findings, there were additional factors that could have been confounding, such as patient co-morbidities, which could not be assessed in this study. In addition, the majority of patients receiving LDLT were pediatric patients. We did not look at characteristics of wait-listed patients, and that can be further explored. Lastly, the diagnosis of ALF status 1 is based on diagnostic codes, and may have been miscoded.

## 5. Conclusions

LDLT remains a minority of all LTs in the US. There were improved 1- and 5-year survival rates among adult and pediatric patients combined, LDLT and DDLT recipients with ALF, and no difference in survival rates in the pediatric population. Overall, there was a trend for better survival of pediatric patients compared to adult LT recipients with ALF who underwent LDLT and DDLT.

## Figures and Tables

**Table 1:** Clinical characteristics of LDLT and DDLT cases in pediatric LT recipients with ALF.

Patient Characteristics (n = 1619)	LDLT (n = 164, 10.1%)	DDLT (n = 1455, 89.9%)	*p*-Value
Age	3 (1–6)	4 (1–12)	<0.001
Gender, female, n (%)	62 (37.8%)	668 (45.9%)	0.04
Blood type			0.26
O	96 (58.5%)	754 (51.8%)	
A	46 (28.0%)	474 (32.5%)	
B	20 (12.2%)	182 (12.5%)	
AB	2 (1.2%)	45 (3.1%)	
Race			0.02
White	95 (57.9%)	664 (45.6%)	
African American/Black	19 (11.6%)	254 (17.5%)	
Hispanic	41 (25.0%)	387 (26.6%)	
Asian	7 (4.3%)	84 (5.8%)	
Other	2 (1.1%)	66 (4.5%)	
Etiology			0.11
Hepatitis A	1 (0.1%)	1 (0.6%)	
Hepatitis B	0 (0.0%)	2 (0.1%)	
DILI	4 (2.4%)	62 (4.3%)	
Wilson disease	5 (3.1%)	87 (6.0%)	
Biliary atresia	10 (6.1%)	110 (7.6%)	
Other	64 (39.0%)	458 (31.5%)	
Unknown	80 (48.8%)	735 (50.5%)	
Sodium (mmol/L)	140.3 (138–141)	140.3 (138–142)	0.40
Creatinine (mg/dL)	0.4 (0.2–0.7)	0.4 (0.3–0.9)	0.01
Bilirubin (mg/dL)	17.8 (10.3–23.1)	15.7 (7.3–22.7)	0.07
INR	3 (2–4.3)	2.6 (1.7–3.8)	0.007
Dialysis	10 (6.1%)	182 (12.5%)	0.02
Ascites	57 (31.7%)	569 (39.1%)	<0.001
Hepatic encephalopathy	51 (31.1%)	3918 (81.9%)	0.04
Wait time (days)	3.5 (2–9)	5 (2–13)	0.01
Donor			
Donor age (years)	32 (26–37)	16 (3–27)	<0.001
Donor gender, female	95 (57.9%)	603 (41.4%)	<0.001
Blood type			0.19
O	122 (74.3%)	1042 (71.6%)	
A	26 (15.8%)	307 (21.1%)	
B	15 (9.1%)	89 (6.1%)	
AB	1 (0.6%)	17 (1.1%)	
BMI	24.4 (22.1–27.0)	20.7 (17.0–24.3)	<0.001
Cold ischemia time (hours)	2.0 (1.0–4.7)	7 (5.9–9)	<0.001

BMI: body mass index, DILI: drug-induced liver injury. Reference ranges: sodium: 135–145 mmol/L, creatinine: 0.50–1.10 mg/dL, bilirubin: 0.3–1.2 mg/dL, INR: 0.8–1.1. Categorical variables are reported as counts and percentages and continuous variables are reported as medians with interquartile ranges.

**Table 2:** Clinical characteristics of LDLT and DDLT cases in adult LT recipients with ALF.

Patient Characteristics (n = 3340)	LDLT (n = 16, 0.5%)	DDLT (n = 3324, 99.5%)
Age	34.5 (21–49.5)	40 (29–53)
Female gender	14 (87.5%)	2258 (67.9%)
Blood type		
O	9 (56.2%)	1553 (46.7%)
A	4 (25.0%)	1147 (34.5%)
B	3 (18.7%)	475 (14.2%)
AB	0 (0.0%)	149 (4.4%)
Race		
White	10 (62.5%)	1895 (57.0%)
African American/Black	3 (18.7%)	687 (20.6%)
Hispanic	2 (12.5%)	383 (11.5%)
Asian	1 (6.2%)	287 (8.6%)
Other	0 (0.0%)	72 (2.1%)
Etiology		
Hepatitis A	1 (6.2%)	58 (1.7%)
Hepatitis B	0 (0.0%)	259 (7.7%)
DILI	3 (18.7%)	753 (22.6%)
Wilson disease	1 (6.2%)	222 (6.6%)
Biliary atresia	0 (0.0%)	0 (0.0%)
Other	6 (37.5%)	1435 (43.1%)
Unknown	5 (31.2%)	597 (17.9%)
Sodium (mmol/L)	140.3 (137–141)	140.3 (137–143)
Creatinine (mg/dL)	1.0 (0.7–2.0)	1.2 (0.8–2.4)
Bilirubin (mg/dL)	5.2 (2.9–17.8)	19.3 (9.2–27.2)
INR	2.2 (1.3–4.6)	3 (2.2–4.3)
Dialysis	2 (12.5%)	758 (22.8%)
Ascites	6 (37.5%)	1877 (56.4%)
Hepatic encephalopathy	14 (87.5%)	3075 (92.5%)
Wait time (days)	3.5 (2–117)	2 (2–4)
Donor characteristics		
Donor age (years)	45 (33.5–50)	36 (22–50)
Donor gender, female	8 (50.0%)	1368 (41.1%)
Blood type		
O	11 (68.7%)	2483 (74.7%)
A	4 (25.0%)	680 (20.4%)
B	1 (6.2%)	143 (4.3%)
AB	0 (0.0%)	18 (0.5%)
BMI	24.8 (22.6–27.5)	25.1 (22.3–28.2)
Cold ischemia time (hours)	1.7 (1–4.8)	6.2 (5–7.5)

BMI: body mass index, DILI: drug-induced liver injury. Reference ranges: sodium: 135–145 mmol/L, creatinine: 0.50–1.10 mg/dL, bilirubin: 0.3–1.2 mg/dL, INR: 0.8–1.1. Categorical variables are reported as counts and percentages and continuous variables are reported as medians with interquartile ranges.

## Data Availability

Data can be requested at: https://unos.org/data/.

## Abbreviations

ALF	Acute liver failure
DDLT	Deceased donor liver transplantation
DILI	Drug-induced liver injury
LT	Liver transplantation
LDLT	Living donor liver transplant
OPTN	Organ Procurement and Transplantation Network
UNOS	United Network for Organ Sharing
US	United States
